# Assessment of Cooking Matters Facebook Platform to Promote Healthy Eating Behaviors among Low-Income Caregivers of Young Children in the United States: A Pilot Study

**DOI:** 10.3390/nu13082694

**Published:** 2021-08-04

**Authors:** Qi Zhang, Jill Panichelli, Leigh Ann Hall

**Affiliations:** 1 School of Community and Environmental Health, Old Dominion University, Norfolk, VA 23529, USA; 2 Share Our Strength, 1030 15th St NW, Suite 1100W, Washington, DC 23005, USA; jpanichelli@strength.org (J.P.); lahall@strength.org (L.A.H.)

**Keywords:** social media, young child, healthy food, low income

## Abstract

How best to deliver healthy-eating education through social media among a low-income population remains understudied. To assess the impact of the Cooking Matters (CM) Facebook page on healthy eating behaviors among low-income caregivers, we conducted a pre–post survey of new CM Facebook followers in early 2020. A convenience sample was recruited at baseline from WICShopper app users and the CM Facebook page. The recruited sample included 397 low-income caregivers of a child younger than 6 who never followed CM Facebook. Among the baseline caregivers, 184 completed the follow-up survey. Paired t-test and McNemar–Bowker tests were conducted to compare the outcomes pre- and post-following CM Facebook. A binary indicator was developed to measure whether the outcomes were improved (1 = Improved; 0 = Not improved). Multi-variable logistic regressions were applied to examine the relationship between whether the outcome was improved with reference to the baseline socio-demographics. No significant differences were detected between pre and post outcomes overall (*p* > 0.05), except improvement in feeding healthy meals within the budget available (*p* < 0.05). However, improvement in select outcomes was more significant in men and single-parent households. The CM Facebook page could be an important platform to influence low-income caregivers of young children.

## 1. Introduction

Healthy eating behaviors in early childhood are important determinants of food preference and dietary quality in later stages of life, which can be linked with a decreased risk for obesity, hypertension, cardiovascular diseases, and other diseases [1] However, low-income children in the U.S. suffer disproportionally from poor dietary quality [2]. Given the synergetic correlations between caregivers’ and children’s dietary behaviors, it is important to implement effective interventions in early childhood among low-income families [3]. Multiple barriers in the food environment, such as access to food resources, and misconceptions about what qualifies as healthy foods or the belief that persistent eating can reduce hunger prevent low-income caregivers from adopting the appropriate healthy eating behaviors in their households [4,5]. Therefore, how to deliver healthy-eating education effectively to these caregivers is important in research and practice. 

Social media is a promising platform for disseminating health information and promoting healthy eating among users, including low-income populations [6,7,8]. The advantages are that it can overcome physical barriers, e.g., lack of public transportation in rural areas, in delivering such information to targeted groups and requires relatively little time and money [9]. However, given numerous social media options available to promote healthy behaviors and limited time the target groups can spend on social media on a daily or weekly basis, it is challenging for a single social media site to capture and keep the attention of their target groups [10]. Therefore, health educators need to choose appropriate platforms to promote healthy behaviors—namely, platforms that are reaching a significant proportion of the audience they wish to reach and where this audience is spending more time. 

Facebook is still one of the most-used social media platforms among U.S. adults, 69% of whom used Facebook in 2019, second to YouTube (73%), and followed by Instagram as a distant third (37%) [11]. Among social media users, Facebook has the highest prevalence of daily users (74%), followed by Instagram (63%), Snapchat (61%), and YouTube (51%). Among adults with household incomes lower than $30,000, 69% of them used Facebook on a daily basis [12]. Another national survey indicated that two-thirds of mothers received information and half of mothers obtained emotional or social support for parenting issues through Facebook [13]. Because of this ubiquitous use, the platform has been used for various interventions targeting mothers [14,15]. However, little is known about how Facebook can impact low-income mothers or other caregivers with young children to promote healthy eating at home. 

Cooking Matters (CM) is a national program of Share Our Strength to provide low-income caregivers with essential skills in shopping for and cooking healthy meals via hands-on cooking classes, grocery store tours, and digital media [16]. According to social cognitive theory, the learning experience in CM’s face to face classes leads to improved dietary behaviors, such as more fruit intake, and better self-efficacy in terms of food preparation and food resource management [17,18]. Interviews with low-income caregivers who used an independent CM phone application (app) indicated the recipe catalogue inspired them to make healthy meals for the family, although the number of CM app users was much smaller than the number of CM Facebook followers (>22,000) [18]. The CM Facebook page regularly publishes seasonal recipes, tips for food planning and cooking, related videos, and live events. CM social posts focus on easy, healthy recipes to prepare on a limited budget, live events that include recipe and skill demos from experienced CM instructors, and a variety of videos addressing caregivers’ barriers to healthy eating and encouraging positive behaviors like meal planning, involving kids in the kitchen, and making healthy foods available for snacking. However, the impact this Facebook page has on the food-related behaviors of low-income caregivers of young children (≤5 years) is unknown. Given the popularity of Facebook and the restricted opportunities for off-line activities during the COVID-19 pandemic, assessment of this question could generate important results to shape CM’s future interventions and provide information for other stakeholders about how to adopt or improve Facebook as a platform to promote healthy eating. To achieve this goal, CM implemented a pilot study in 2020.

## 2. Methods

### 2.1. Study Design

This study adopted a single group pre–post design to evaluate the food-related behaviors and outcomes among new followers of the CM Facebook page who were low-income caregivers of children age 5 or younger. To be eligible for the baseline survey, participants had to satisfy the following inclusion criteria: a. Never followed the CM Facebook page before; b. Caregiver of a child age 5 years or younger and provides food, including meal planning, shopping, and cooking for the child; c. Household income below or equal to 185% of the federal poverty threshold in 2019 [19]; d. “Very likely” or “likely” to participate in the follow-up survey in 2 months. The income threshold used in c. was consistent with the income eligibility requirements of the Special Supplemental Nutrition Program for Women, Infants, and Children (WIC) [20]. The follow-up criterion of d. was included to increase the participation rate in the follow-up survey. The baseline survey provided a link to the CM Facebook page and asked the respondents to review it and answer how likely they would be to follow the page. Participants unlikely to follow the page were not included in the study. At the end of the baseline survey, the respondents were required to provide their cell phone numbers so a $10 Amazon gift card could be delivered after completion. The baseline survey was started in early February 2020 and ended in early March 2020. After 2 months, a follow up survey link was delivered to the same cell phone number. Another $10 Amazon gift card was sent after the follow-up survey was completed. Only new CM Facebook followers were included in the pre–post comparison. 

To recruit the participants, the baseline survey link was placed in multiple locations: a recruitment banner was set up on the JPMA WICShopper app for Kansas and Florida participants, and a recruitment link was posted on the CM Facebook page for referral of new followers. WICShopper is the standard app to assist WIC benefit redemption and program participation for WIC participants in Kansas and Florida. The WICShopper app was only used as a recruitment platform to the CM Facebook page in this study. This study was approved as exempt by the Institutional Review Board at Old Dominion University (Approval #: 1492932). 

### 2.2. Measurement

Outcomes were measured from baseline and follow-up surveys with questions about food eating behaviors, attitudes toward cooking, and self-confidence in food planning and preparing healthy food for the participants’ households. The food eating behavior questions were focused on the frequency with which the oldest child in the household who was five years old or younger ate fruits and vegetables. The question on attitudes toward cooking included “cooking takes too much time,” “cooking is frustrating,” and “it is too much work to cook.” These questions were adopted from the CM for Adults (CMA) survey, which was developed and assessed by the Gretchen Swanson Center for Nutrition [17,21]. 

Self-confidence in performing the following activities was measured in Likert scale: feeding the family healthy food with the money available, providing healthy drinks to the family, handling meal-time frustrations with child(ren) in the family, and making mealtime a positive experience for the respondent and the child(ren) in the family. These self-efficacy questions were pilot-tested and assessed by IMPAQ International, LLC, in a convenience sample of 757 low-income caregivers of children age 5 or younger. The preliminary results of the assessment indicated high internal consistency, with a Cronbach’s alpha = 0.87. 

To measure the exposure to CM Facebook content, new followers were asked about their frequency viewing the content, their interaction with the contents (including reacting to posts [e.g., “liking” them], comments on posts, sharing posts, watching Facebook Live, and watching videos), and the content they liked. Finally, the survey asked Likert scale questions about how much the CM Facebook page had changed their food planning, shopping, cooking, and feeding behaviors, as well as their overall life. 

Socio-demographics were controlled in the analyses. These measures were self-reported by caregivers, including sex, age group (18–24, 25–29, 30–34, 35–39, 40 or older), education (less than high school, high school diploma or GED, some college but no degree, associate degree, bachelor’s degree or above), race/ethnicity (non-Hispanic White, non-Hispanic African American, Hispanic, Other), number of adults (1, 2, 3+) and children in the household (1, 2, 3+). 

### 2.3. Statistical Analyses

Descriptive statistics were provided for the outcome variables in the baseline and follow-up surveys, CM Facebook page activities and opinions, and socio-demographics. Pearson’s chi-squared goodness of fit tests were applied to compare the socio-demographics between participants who completed the follow-up surveys and those who did not. Paired-sample t-tests and McNemar–Bowker tests were applied to test the significance of the differences between continuous, e.g., timing, and categorical outcome variables, e.g., frequencies. Changes in eating behaviors, attitudes towards cooking, and self-efficacy in healthy eating between the baseline and the follow-up surveys were coded as binary: 1 = improvement, 0 = no change or worse based on the Likert scale in the baseline. The dichotomized outcomes can help understand what socio-demographics were associated with the improvement outcomes among new CM Facebook followers. Logistic regression was applied to examine the relationship between whether the outcome was improved and the baseline socio-demographics [22]. Odds ratios (OR) of socio-demographics and 95% confidence intervals were reported to measure the ratios of the odds of the outcome improvement in certain socio-demographic groups compared with the reference group. An OR > 1, OR = 1, or OR < 1 indicated that that socio-demographic group had higher, equal, or lower odds of outcome improvement than the reference group, respectively. *p* < 0.05 was significant, with Bonferroni adjustment to account for multi-comparison testing. Stata 14 was used for analyses (StataCorp LLC, College Station, TX, USA). The corresponding author has the full access to the data used in this study, which is available upon request. 

## 3. Results

Table 1 presents the socio-demographics of the respondents in the baseline survey (*N* = 397) and tests the potential difference between respondents (*n* = 184) and non-respondents (*n* = 213) in the follow-up survey. Almost 85% of the respondents were recruited from the WICShopper app. Although 13.6% of the participants in the baseline survey were male, 21.7% of the follow-up survey participants were male, which was significantly more than the 6.6% of males who did not participate in the follow-up survey (*p* < 0.001). More respondents from younger age groups participated in the follow-up survey (*p* < 0.001). Approximately 2/3 of respondents in the baseline survey had received some education at the college level or above. However, 3/4 of the respondents in the follow-up survey had received a similar level of education, i.e., college level or above, significantly more than the non-participants in the follow-up survey (*p* < 0.001). No significant difference was detected in the racial/ethnic composition between follow-up survey participants and non-participants. Approximately 15.6% of baseline survey respondents had 1 adult in the household, but 8.2% of the follow-up survey participants had the same number of adults in their households, which suggests that households with single parents were less likely to participate in the follow-up survey (*p* = 0.001). 

Among the participants in the follow-up survey (*n* = 184), 17 respondents reported that they did not follow the CM Facebook page. Therefore, the pre–post comparison was limited to 167 (90.7%) new CM Facebook followers for 2 months. Table 2 compares the food-related behaviors, attitudes toward cooking, and perceptions of self-efficacy about providing healthy food in the households in the baseline and follow-up surveys; the results indicated no statistically significant difference between the outcomes, except feeding healthy meals within budget (*p* = 0.02). Table 3 and Table 4 present the regression results showing which groups were more or less likely to be improved in these outcomes. Women were less likely to improve their attitudes toward cooking (“cooking is frustrating”) over the 2 months’ follow-up period (OR = 0.21, 95% CI = 0.07–0.65) than men. Compared with multi-adult families, single parent families were more likely to have improved attitudes toward cooking (“too much work to cook”). Caregivers from older age groups (35–39) were less likely to achieve the improvement of preparing healthy meals with the time available (OR = 0.02; 95% CI = 0.001–0.31) than caregivers from younger age groups. 

About 3/4 of new followers viewed the CM page at least a few times a week (Table 5). Interaction with the CM Facebook page varied from 28.7% of followers that commented on posts to 59.3% of followers who reacted to the posts (e.g., like). The CM recipes were the most-liked content on the Facebook page among the new followers, followed by CM tips on feeding children and tips on food planning. In addition, participants reported the impact of the CM’s Facebook page on their food-related behaviors, attitudes, perceptions of self-efficacy, and overall life. Consistently, around 1/3 of the respondents rated CM impacting them “a great deal,” and approximately 1/4 of the respondents rated the impact as “somewhat.” Therefore, for new followers, CM had become an important information source that had helped change their healthy eating behaviors and attitudes. 

## 4. Discussion

This is one of the first quantitative studies that has examined using Facebook as a platform to promote healthy eating among low-income caregivers of young children. Given the popularity of Facebook in the caregivers’ generation, food and health organizations could take advantage of this important platform for nutrition education and health promotion [23]. This study demonstrated that more than 90% of respondents in the baseline survey still followed the CM Facebook page after 2 months, while more than half of them thought the page impacted their food-related activities and overall life. Moreover, new followers actively interacted with various components of the CM Facebook page. Therefore, the CM Facebook page offers opportunities to promote healthy food eating among low-income caregivers of young children. 

The overall insignificant changes in outcomes were consistent with the literature that has documented barriers for behavioral changes among low-income caregivers [24]. The general positive attitudes towards cooking were consistent with the results found in CM app users [18]. Notably, in-person CM classes can achieve significant improvement in more outcomes, including food resource management and self-efficacy in cooking and preparing healthy meals [16]. However, this pilot study indicated that the improvement in healthy eating activities or attitudes were significant in men and in single-parent households, all of whom could have unhealthier eating behaviors compared to their counterparts. The findings of this study indicate that Facebook could be a more targeted platform ideally suited for those groups who are traditionally less responsive to health promotions, although more research is needed to validate this hypothesis. Moreover, the results need to be interpreted carefully in view of a few limitations, including this study’s small sample size, single group pre–post design, and reliance on self-reported results. In addition, income information was not solicited since it was challenging to solicit high-quality data about this in an anonymous online survey. Moreover, the feedback from participants is mainly subjective, which can make the results over-optimistic. More objective study can be explored in future research to examine the actual food intake before and after following the Facebook page. However, these limitations do not reduce the significance of this pilot study on how CM’s Facebook site may play a role in nutrition education and health information dissemination among low-income caregivers of young children. 

## 5. Implications for Research and Practice

CM is a national campaign to give caregivers the skills they need to regularly serve nutritious foods to their young children. The CM Facebook page has been demonstrated to be a platform with high and consistent rates of use among this target group. It thus provides important opportunities to disseminate healthy food information and cooking tips that may change behaviors among followers. Social media may serve as a platform to promote healthy eating among low-income children, which is an important objective in Healthy People 2030 [25]. 

## Figures and Tables

**Table 1:** Descriptive statistics of caregivers in baseline surveys and by follow-up status (*N* = 397).

	Total % (*N* = 397)	Follow-up % (*n* = 184)	Non-Follow-Up % (*n* = 213)	*p*-Value ^a^
**Recruitment from**				
WICShopper App	84.6	81.5	87.3	0.11
Non-WICShopper App	15.4	19.5	12.7	
Sex				
Female	86.4	78.3	93.4	<0.001
Male	13.6	21.7	6.6	
**Age group**				
18–24	12.6	7.6	16.9	<0.001
25–29	35.3	44	27.7	
30–34	28.0	31.5	24.9	
35–39	13.4	10.9	15.5	
40 or above	10.8	6	15	
**Education**				
<High School	5.8	1.6	9.4	<0.001
High School or GED	29.5	23.9	34.3	
Some college, no degree	28.2	30.4	26.3	
Associate degree	23.2	33.2	14.6	
Bachelor’s degree or above	13.3	10.9	15.5	
**Race/ethnicity**				
Non-Hispanic white	44.3	40.8	47.4	0.16
Non-Hispanic black	13.4	15.2	11.7	
Hispanic	37.3	40.8	34.3	
Other	5.0	3.3	6.6	
**Number of adults in household**				
1	15.6	8.2	22.1	0.001
2	48.9	53.3	45.1	
3 or more	35.5	38.6	32.9	
**Number of children in household**				
1	44.6	51.1	39	0.05
2	31.2	28.3	33.8	
3 or more	24.2	20.7	27.2	

^a^ Pearson’s Chi-square goodness-of-fit test for categorical variables between caregivers participating in the follow-up survey and those not participating in the follow-up survey. Bonferroni adjusted significance level: *p* < 0.007.

**Table 2:** Food-related behaviors and attitudes in baseline and follow-up surveys among new Cooking Matters Facebook followers (*n* =167).

	Baseline (Mean or %)	Follow up (Mean or %)	*p*-Value ^a^
**Fruit frequency (child ≤ 5 year)**			0.86
Not at all	1.2	0.0	
Once a week	2.5	2.4	
More than once a week	14.2	13.3	
Once a day	31.5	33.3	
More than once a day	50.6	50.9	
**Veg frequency (child ≤ 5 year)**			0.29
Not at all	1.2	0.6	
Once a week	7.4	6.7	
More than once a week	21.0	25.5	
Once a day	27.2	30.3	
More than once a day	43.2	37.0	
**Attitude towards cooking (1: Strongly agree; 7: Strongly disagree)**			
Cooking takes too much time	4.2	4.3	0.18
Cooking is frustrating	4.9	4.9	0.91
Too much work to cook	4.7	4.6	0.52
**Food-related self-efficacy (1: Not confident at all; 5: Very confident)**			
Feed healthy meal within the budget	4.0	4.2	0.02
Provide healthy drink	4.2	4.3	0.15
Handle meal-time frustrations	4.1	4.1	0.23
Positive meal experience with children	4.4	4.3	0.18
Prepare healthy meal with time available	4.2	4.3	0.45

^a^ McNemar–Bowker test for categorical variables in the baseline and in the follow-up surveys; Paired *t*-test for continuous variable in the baseline and in the follow-up surveys; Bonferroni adjusted significance level: *p* < 0.005.

**Table 3:** Logistic regression results of improvement in eating behaviors and attitudes towards cooking among new Cooking Matters Facebook followers (*n* = 167).

	Fruit Frequency		Veg Frequency		Cooking Takes too Much Time		Cooking is Frustrating		Too Much Work to Cook	
	OR ^a^	95% CI ^b^	OR	95% CI	OR	95% CI	OR	95% CI	OR	95% CI
**Female**	1.16	0.29–4.59	2.76	0.50–15.33	0.4	0.14–1.13	0.21	0.07–0.65 ^c^	0.25	0.08–0.73
**Age group (Reference group: 18–24)**										
25–29	0.42	0.07–2.38	0.61	0.08–4.68	0.29	0.04–2.02	11.46	1.51–87.20	0.99	0.15–6.63
30–34	0.48	0.09–2.43	0.91	0.13–6.11	0.22	0.03–1.45	7.27	0.99–53.27	0.95	0.15–5.92
35–39	0.06	0.03–1.33	0.46	0.04–5.66	0.4	0.04–3.97	8.7	0.75–101.36	2.1	0.25–17.94
40 or above	0.15	0.01–2.22	1.2	0.10–13.91	0.3	0.02–4.09	5.27	0.42–66.63	1.0	N/A ^d^
**Education (Ref group: <High school)**										
High School or GED	0.06	0.003–1.13	1.17	0.17–7.94	0.06	0.003–1.43	4.24	0.29–62.21	1.12	0.07–17.93
Some college	0.05	0.003–0.80	1.94	0.33–11.44	0.21	0.01–3.67	0.65	0.05–8.80	0.77	0.05–11.03
Associate degree	0.06	0.003–1.01	1.62	0.27–9.85	0.36	0.02–6.56	1.37	0.10–18.70	1.72	0.12–24.67
Bachelor’s degree or above	0.06	0.003–1.41	1	N/A ^d^	0.09	0.004–2.11	1.12	0.07–18.43	0.68	0.04–11.93
**Race/ethnicity (Ref group: Non-Hispanic white)**										
Non-Hispanic black	0.92	0.25–3.33	1.12	0.27–4.62	6.85	1.87–25.12	3.07	0.96–9.85	1.21	0.34–4.35
Hispanic	0.68	0.21–2.25	1.47	0.46–4.73	5.3	1.64–17.07	1.21	0.39–3.74	1.28	0.40–4.03
Other	0.8	0.07–8.83	0.7	0.06–8.20	7.01	1.06–46.49	2.2	0.28–17.55	3.50	0.45–27.46
**# of adults (Ref group: *N* = 1)**										
2	0.83	0.18–3.89	3.35	0.38–29.93	0.51	0.10–2.53	0.57	0.14–2.35	0.17	0.04–0.74
3 or more	1.1	0.17–6.90	1.87	0.16–21.08	0.63	0.11–3.61	0.35	0.07–1.82	0.10	0.02–0.56 ^c^
**# of children (Ref group: *N* = 1)**										
2	1.58	0.52–4.81	1.61	0.52–5.04	1.57	0.58–4.28	4.06	1.48–11.12	0.75	0.26–2.19
3 or more	2.57	0.65–10.12	1.47	0.38–5.76	3.87	0.99–15.19	0.75	0.19–2.92	0.61	0.17–2.20

^a^ OR: Odds ratios; ^b^ CI: Confidence interval: ^c^ Bonferroni’s adjusted significance level: *p* < 0.01; ^d^ Collinearity.

**Table 4:** Logistic regression of improvement in self-efficacy in healthy eating among new Cooking Matters Facebook followers (*n* = 167).

	Feed Healthy Meal within the Budget		Provide Healthy Drink		Handle Meal-time Frustration		Positive Meal Experience with Children		Prepare Healthy Meal with Time	
	OR ^a^	95% CI ^b^	OR	95% CI	OR	95% CI	OR	95% CI	OR	95% CI
**Female**	0.78	0.27–2.21	1.1	0.86	0.91	0.29–2.86	0.75	0.24–2.40	0.38	0.12–1.28
**Age group (Reference group: 18–24)**										
25–29	0.69	0.15–3.15	1.54	0.29–8.17	4.08	0.63–26.36	2.83	0.44–18.25	0.23	0.04–1.24
30–34	0.68	0.16–2.78	1.57	0.32–7.61	3.49	0.61–20.06	2.06	0.35–12.26	0.17	0.03–0.85
35–39	0.72	0.11–4.66	2.49	0.34–18.36	2.04	0.23–18.17	1.03	0.09–11.33	0.02	0.001–0.31 ^c^
40 or above	1.04	0.15–7.03	4.54	0.59–34.92	2.06	0.20–20.78	0.75	0.05–11.27	0.4	0.05–3.26
**Education (Ref group: <High school)**										
High School or GED	0.78	0.05–11.38	0.79	0.05–12.42	0.5	0.12–2.02	1.47	0.30–7.17	1.03	0.20–5.25
Some college	1.33	0.10–17.87	0.3	0.06–11.93	0.4	0.11–1.49	1.21	0.27–5.41	5.25	1.02–26.92
Associate degree	0.68	0.05–9.37	0.65	0.04–9.55	0.58	0.16–2.14	1.1	0.25–4.94	2.96	0.57–15.29
Bachelor’s degree or above	0.85	0.05–13.24	0.71	0.04–11.69	1	N/A ^d^	1	N/A ^d^	1	N/A ^d^
**Race/ethnicity (Ref group: Non-Hispanic white)**										
Non-Hispanic black	0.62	0.20–1.86	0.85	0.28–2.55	0.94	0.29–3.06	1.25	0.37–4.24	1.84	0.56–6.10
Hispanic	0.6	0.22–1.60	0.49	0.17–1.36	1.08	0.38–3.03	0.84	0.28–2.53	0.71	0.22–2.27
Other	3.47	0.51–23.40	0.41	0.04–4.19	0.39	0.03–4.45	1.57	0.23–10.69	0.58	0.07–5.07
**# of adults (Ref group: *N* = 1)**										
2	0.69	0.20–2.38	0.71	0.20–2.58	1.25	0.32–4.90	2.17	0.42–11.26	0.75	0.19–2.91
3 or more	0.64	0.15–2.81	1.25	0.28–5.55	0.45	0.09–2.35	1.33	0.20–8.72	0.35	0.07–1.87
**# of children (Ref group: *N* = 1)**										
2	0.63	0.25–1.60	0.48	0.18–1.30	2.05	0.78–5.39	1.17	0.42–3.25	0.59	0.20–1.73
3 or more	1.24	0.42–3.69	0.9	0.29–2.76	1.21	0.37–3.90	1.37	0.39–4.76	4.22	1.21–14.72

^a^ OR: Odds ratio; ^b^ CI: Confidence interval; ^c^ Bonferroni’s adjusted significance level: *p* < 0.01; ^d^ Collinearity.

**Table 5:** Exposure to Cooking Matters (CM) Facebook content among followers (*n* = 167).

Exposure	%	SE
**Frequency viewing CM Facebook content**	
One or more times each day	24.6	3.3
A few times each week	40.7	3.8
A few times each month	12.6	2.6
Once or twice each month	9.6	2.3
Never or almost never	12.6	2.6
**Interaction with CM Facebook content**		
Reacting to posts (e.g., like)	59.3	3.8
Comment on posts	28.7	3.5
Sharing posts	37.1	3.7
Watching Cooking Matters Live	40.1	3.8
Watching Cooking Matters videos	48.5	3.9
**Liked CM contents**		
Photos	38.3	3.8
Videos	56.3	3.8
Recipes	74.3	3.4
Tips on food planning	53.9	3.9
Tips on grocery shopping	32.3	3.7
Tips on feeding your children	59.9	3.8
Facebook Live	15.0	2.8

## Data Availability

The data is available upon request.

## Abbreviations

CM	Cooking Matters
WIC	the Special Supplemental Nutrition Program for Women, Infants, and Children.
